# Polycyclic Aromatic Hydrocarbons in Sediments and Bivalves on the Pacific Coast of Japan: Influence of Tsunami and Fire

**DOI:** 10.1371/journal.pone.0156447

**Published:** 2016-05-27

**Authors:** Mayu Onozato, Atsuko Nishigaki, Kenji Okoshi

**Affiliations:** Department of Environmental Science, Faculty of Science, Toho University, Funabashi, Chiba, Japan; University of California, Merced, UNITED STATES

## Abstract

Surface sediments and at least one edible bivalve species (*Ruditapes philippinarum*, *Mytilus galloprovincialis*, and *Crassostrea gigas*) were collected from each of seven intertidal sites in Japan in 2013. The sites had experienced varying levels of tsunami and fire disturbance following the major earthquake of 2011. Eight polycyclic aromatic hydrocarbons (PAHs) were identified and analyzed by gas chromatography–mass spectrometry. Total sediment PAH concentration (*C*_T_), the sum of the average concentrations of the eight PAHs, was 21–1447 μg kg^-1^-dry. Relative to the average level of one type of PAH in sediments collected around Japan in 2002 (benzo[*a*]pyrene = 21 μg kg^-1^-dry), five of the seven sites showed concentrations significantly lower than this average in 2013. The *C*_Ts_ for the three bivalves (134–450 μg kg^-1^-dry) were within the range of the previous reports (2.2–5335 μg kg^-1^-dry). The data suggest that the natural disaster did not increase PAH concentrations or affect the distribution within sediment or bivalves in Tohoku district. Although PAH concentrations at the sites pose no risk to human health, the findings highlight that the observed PAH levels derive from pre- rather than post-quake processes.

## Introduction

Polycyclic aromatic hydrocarbons (PAHs), having two or more fused benzene rings, are a group of organic pollutants that occur widely in the environment. They are recognized as mutagenic and carcinogenic compounds; benzo[*a*]pyrene and benzo[*a*]anthracene are typical carcinogens. Additionally, some PAHs such as benzo[*a*]pyrene are listed as endocrine disruptors in the “environmental hormone” category [1, 2]. Some PAHs are formed due to the incomplete combustion of materials, and are released into the atmosphere by processes such as petroleum and coal combustion [3–5], volcanic eruptions [6], and forest fires [6]. Aquatic environments are also polluted with PAHs through anthropogenic activities such as accidental oil spills, discharge from routine tanker operations, and municipal and urban runoff. Additionally, they tend to accumulate preferentially in river and marine sediments rather than in air or water, due to their high hydrophobicity [7].

The Great East Japan Earthquake of 11 March 2011 was a magnitude 9-class earthquake and the largest of its kind in Japan since the Industrial Revolution. The earthquake epicenter was off the Pacific coast of the Tohoku district in northern Japan, and triggered extremely destructive tsunami waves along the east coast of four prefectures of Tohoku district and strong shaking ac4ross extensive areas of eastern Japan. In tsunami-devastated regions, sediments and marine life along the coast may be at risk of exposure to organic pollutants, including PAHs that have been re-suspended from benthic sediments by the disturbance. Furthermore, in Kesennuma, the tsunami destroyed oil storage tanks, resulting in an oil spill of approximately 11.5 million liters that spread into inundated areas, resulting in major fires. Similar disasters occurred in several enclosed coastal seas of the district. The distribution of pollutants in coastal areas, especially those that were severely damaged, has been monitored intensively following the tsunami. Researchers have demonstrated that PAH concentrations in sediments and bivalves increased after the tsunami [8, 9]. Conversely, PAH levels in the sediments of Gamo Lagoon (Sendai, Miyagi Prefecture) indicated that petroleum contamination was quite low [10]. The tsunami caused extensive damage across the Tohoku region, including to marine life. Bivalves present in the sediment were ejected to the surface by the jet of water and strong wave action. The tsunami and other post-quake phenomena such as liquefaction resulted in immediate damage to coastal marine life [11–14]. However, this was the first earthquake to cause major anthropogenic pollution [13] resulting from the damage inflicted on coastal industrial infrastructure and property, where significant inputs from ship fires and damaged oil tanks resulted in an uncontrolled release of fuel and combustion products into the sea.

**Fig 1:**
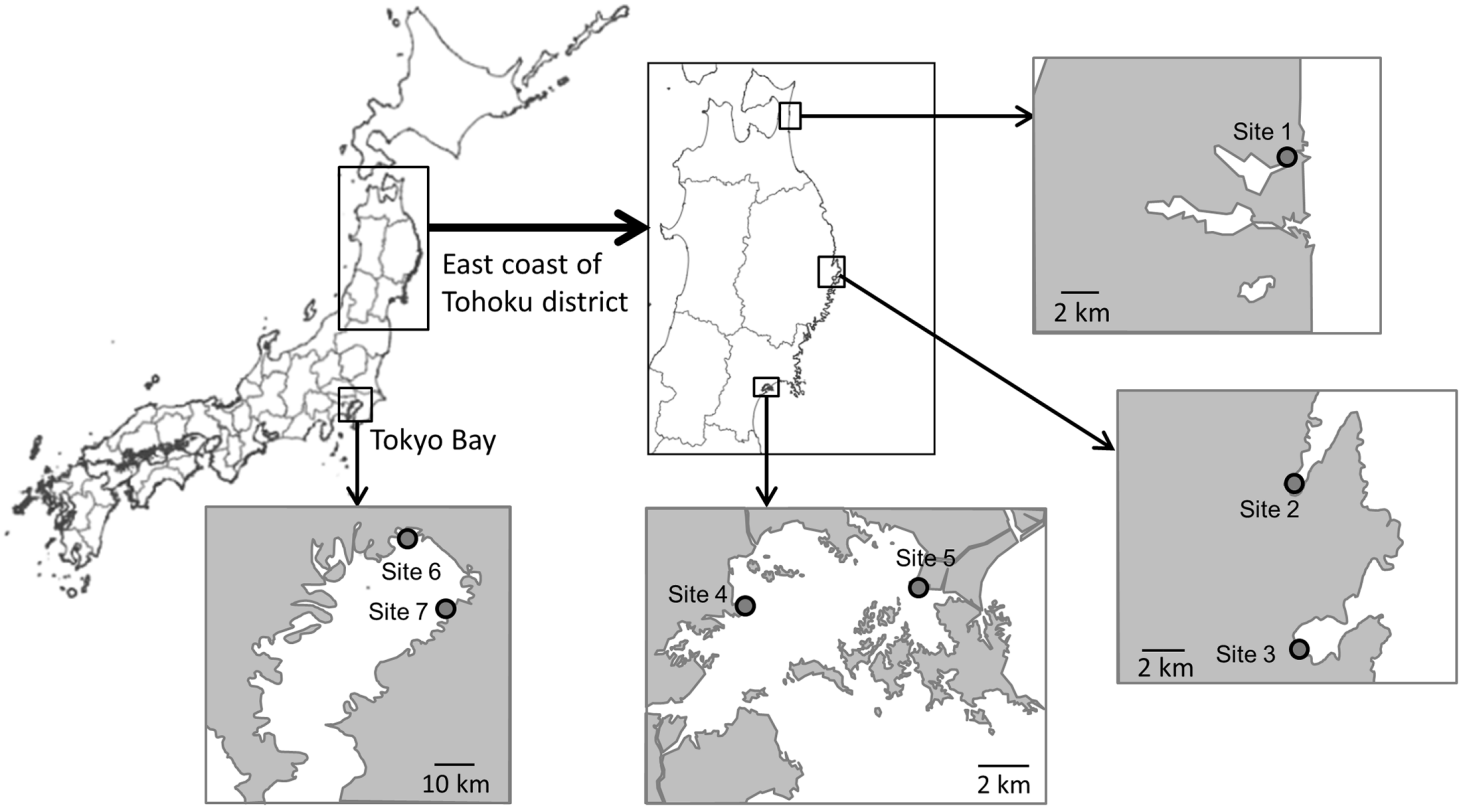
Location of sampling sites in this study. (Site 1: Lake Obuchinuma; Site 2: Tsugaruishi estuary tideland; Site 3: Orikasa estuary tideland; Site 4: Sokanzan tidal flat; Site 5: Tona coast; Site 6: Sanbanze tidal flat; Site 7: Yoro estuary tideland).

The present study determines the concentrations and compositions of PAHs in sediments at five intertidal sites, from north to south, along the east coast of Tohoku district (Fig 1): Lake Obuchinuma (site 1) (40°57’34”N–141°22”17”E), the Tsugaruishi estuary tideland (site 2) (39°35’19”N–141°56’58”E), the Orikasa estuary tideland (site 3) (39°26’52”N–141°57’46”E), the Sokanzan tidal flat (site 4) (38°21’8”N–141°3’34”E), and the Tona coast (site 5) (38°21’55”N–141°8’32”E). These sites experienced varying degrees of damage from the tsunami and fires triggered by the earthquake. Site 1, in Aomori Prefecture, experienced minimal influence by the tsunami [15]. Conversely, the tsunami effects were catastrophic at sites 2 and 3, in Iwate Prefecture [16, 17], which were inundated to heights of 5.9–12.2 m and 4.5–10.9 m, respectively. Additionally, site 3 was damaged by a fire caused by the seismic activity in this area. Sites 4 and 5 are located in the innermost areas of Matsushima Bay in Miyagi Prefecture (Fig 1). Several islands of various sizes, present in the mouth of the bay [18], acted as a barrier that moderated the impact of the tsunami in Matsushima Bay, resulting in inundation heights of only 2.5–4.7 m [19]. Accordingly, this study investigates PAH concentrations at these sites, which have each suffered differently from the tsunami, and have not previously been investigated following the earthquake. Furthermore, the collection and farming of clams, mussels, and oysters has continued along the coast of Tohoku district, making the evaluation of PAH concentrations in bivalves an important public health issue. For comparison with these sampling sites along the east coast of Tohoku district, we replicated the same analysis for sediment and clams at two sites in Tokyo Bay (Fig 1), at the Sanbanze tidal flat (site 6) (35°40’16”N–139°57’52”E) and the Yoro estuary tideland (site 7) (35°32’22”N–140°4’13”E). These sites were minimally impacted by the tsunami, but the latter was exposed to pollution from a fire that occurred in a neighboring oil refinery, caused by the earthquake. Taking into account the degree and extent of tsunami and fire effects at the sampling sites, we assessed the quality of the sediments based on the concentrations and compositions of PAHs, based on our expectation that sites more heavily impacted by the tsunami and fire would have higher PAH concentrations.

## Materials and Methods

### Sample collection

No special permits were required to perform field surveys since the study sites are not privately owned or legislatively protected. We did not involve protected and/or endangered species in this study.

During September and November 2013, surface sediments at least ten locations (to a maximum depth of 5 mm) were collected at low tide from the intertidal zone by scraping the surface sediment using a plastic spoon [20]. All collected sediment samples were placed in a clean bag, mixed, chilled, and transported to the laboratory. Samples were frozen at –20°C until sample preparation. All sediment collections were performed in the same way at all seven sampling sites (Fig 1 and Table 1).

**Table 1:** Description of sediment sampling sites.

Site	Location	Description	Sample grain facies	Inundation height (m)	Other relevant information
1	Lake Obuchinuma	Brackish lake	Medium grained sand and with a small amount of gravel		
2	Tsugaruishi estuary tideland	Innermost area	Coarse-grained sand with a small amount of gravel	5.9–12.2	
3	Orikasa estuary tideland	Innermost area	Coarse-grained sand	4.5–10.9	Seismic fire
4	Sokanzan tidal flat	Inner bay, west	Fine- to medium-grained sand	2.5–4.7	
5	Tona coast	Inner bay, east	Muddy sand	2.5–4.7	
6	Sanbanze tidal flat	Shallow coastal area (inner bay)	Fine- to medium-grained sand with shells		
7	Yoro estuary tideland	River mouth (middle bay)	Fine- to medium-grained sand		Fire at a neighboring refinery

Bivalves such as clams (*Ruditapes philippinarum* or *Mactra quadrangularis*), mussels (*Mytilus galloprovincialis*), and oysters (*Crassostrea gigas*) were collected at the seven sites during sediment sampling (see Table 2). The shell surfaces were washed with clean seawater at the time of collection, and the bivalves were placed in a clean bag, refrigerated, and transported to the laboratory. Shell length (clam and mussel), shell height (oyster), and total weight were measured for each individual. Soft tissues were then removed from the shells and centrifuged to remove excess water prior to freezing at –20°C until further sample preparation.

**Table 2:** Details of bivalve samples collected for analysis.

Bivalves	Site	*N*	Shell size* (mm)	Total weight (g)
C	4	30	37± 3	13 ± 3
C	5	32	28 ±2	14 ± 3
C	6	38	29 ± 2	6 ± 1
C	7	28	40 ± 2	21 ± 3
M	1	13	52 ± 6	18 ± 7
M	2	12	50 ± 5	14 ± 5
M	3	15	64 ± 4	24 ± 3
O	1	13	51 ± 10	54 ± 29
O	2	16	54 ± 7	38 ± 11

* Shell length of C and M, shell height of O, respectively.

(C: *Ruditapes philippinarum* at sites 4–6 and *Mactra quadrangularis* at site 7; M: *Mytilus galloprovincialis*; O: *Crassostrea gigas*).

### Materials

Hexane, acetone, ethanol, and sodium sulfate (Wako Pure Chemicals Industries, Ltd., Japan) used for the analyses were of residual agricultural chemical grade. Water purified by a Millipore Direct-Q^®^ 3UV water purification system was washed with hexane twice prior to use. Authentic PAH standards (phenanthrene [Phe], anthracene [Anth], fluoranthene [Flu], pyrene [Pyr], chrysene [Chry], benzo[*b*]fluoranthene [[b]flu], benzo[*a*]pyrene [[a]pyr], and perylene [Pery]), deuterated PAHs, and *p*-terphenyl-*d*_14_ were purchased from Wako Pure Chemicals Industries, Ltd. (Japan). Deuterated surrogate standards were used to compensate for PAH losses during analysis.

### Sample preparation

PAHs were analyzed using a method modified from one reported previously [20]. The collected sediment was thawed, mixed, and sieved through a 1-mm mesh. Triplicate wet sediment samples from each site (approximately 10–20 g per sample) were extracted twice with acetone after spiking with a deuterium-labeled PAH surrogate standard mixture (phenanthrene-*d*_10_, anthracene-*d*_10_, fluoranthene-*d*_10_, pyrene-*d*_10_, chrysene-*d*_10_, benzo[a]pyrne-*d*_12_, and perylene-*d*_12_). Thereafter, the acetone extract was concentrated to approximately 20 mL by use of a rotary evaporator. The concentrated extract was added to 50 mL of 1 M KOH/EtOH solution and shaken for 15 hours at room temperature (ca. 25°C) in the dark for alkaline decomposition. Following decomposition, the solution was extracted twice with hexane. The extracted hexane solution was washed with water, dehydrated with anhydrous sodium sulfate, filtered, and concentrated to about 1 mL by a rotary evaporator and N_2_ gas. The concentrated solution was subjected to column chromatography with 5% H_2_O-deactivated silica gel and 100 mL of 1% acetone/hexane solution. The eluate was concentrated and diluted to 1 mL with hexane after the addition of *p*-terphenyl-*d*_14_ to the sample as an internal standard, and was analyzed by gas chromatography–mass spectrometry (GC–MS).

Bivalve samples were prepared using the same procedure used for the sediment samples, with some minor adjustments [20]. The soft parts of bivalves were thawed and then homogenized using a food processor. About 6 g of the homogenized sample (*n* = 3) was decomposed with 50 mL of 1 M KOH/EtOH solution for 15 hours using a magnetic stirrer at room temperature after adding the deuterium-labeled PAH surrogate standard mixture. The samples were then shaken vigorously for 2 hours for complete decomposition. The remaining procedure was the same as that used for sediment samples.

### GC–MS analysis

The GC–MS conditions were the same as those reported previously [20]. Briefly, a GC–MS system (QP2010, SHIMADZU, Japan) equipped with a 30-m Rtx-5MS fused silica capillary column (0.25 mm i.d., coated with a 0.25 μm thick 5% diphenyl-95% dimethyl polysiloxane film; Restek, USA) was used to analyze PAHs. Helium (99.99% purity) was used as a carrier gas. The temperature program was optimized so as to separate each PAH quickly: the initial temperature of 50°C was held for 2 min, then increased at 7°C min^-1^ to 310°C, and held for 10 min. The mass spectrometer was operated in electron impact ionization mode at 70 eV. The interface was kept at 250°C and the ionization source at 200°C. Measurements were performed in the selected ion monitoring mode. Each sample solution was measured by GC–MS twice and the average value was used for evaluation. The individual concentrations of PAHs in sediments and bivalves are given on a dry basis.

## Results

### PAH concentrations in sediment samples

The average concentrations of individual PAHs in the sediments collected at seven sampling sites are shown in Table 3. The total concentration (*C*_T_) of PAHs is defined as the sum of the average concentrations of the eight PAHs, and the value for each sampling site is also shown in the table.

**Table 3:** Average concentrations (μg kg^-1^-dry) of PAHs in sediments (*n* = 3) versus sediment quality assessment guidelines.

	Site 1	Site 2	Site 3	Site 4	Site 5	Site 6	Site 7	ERL	ERM
Phe	6.54	7.95	7.17	24	153	5.33	7.28	240	1500
Anth	0.86	1.52	1.7	8.33	66.8	1.58	1.36	85	1100
Flu	3.79	9.11	8.02	52.8	365	3.76	3.51	600	5100
Pyr	2.83	7.36	6.19	49.3	321	2.95	2.31	670	2600
Chry	1.86	3.92	5.48	24.2	179	2.15	1.69	384	2800
[b]flu	1.58	3.26	4.96	13.4	110	1.95	1.49	-	-
[a]pyr	1.57	3.41	5.64	22.8	177	2.16	2.1	430	1600
Pery	1.47	3.15	1.89	7.53	75.2	4.67	3.35	-	-
Σ PAHs	20.5	39.7	41.1	202.4	1447	24.6	23.1	-	-
Anth / [Anth]+[Phe]	0.116	0.161	0.192	0.258	0.304	0.229	0.157		
[Flu] / [Flu]+[Pyr]	0.573	0.553	0.564	0.517	0.532	0.56	0.603		

Σ PAHs is denoted by *C*_T_ in the text.

Although sites 1–5 are located in the Tohoku district (Fig 1) and sites 6 and 7 in Tokyo Bay, the *C*_T_ values of sites 1, 6, and 7 were almost identical, at 21–25 μg kg^-1^-dry. Sites 2 and 3 show *C*_T_ values approximately double those of sites 1, 6, and 7, at ~40 μg kg^-1^-dry. The *C*_T_ values for sites 4 and 5 were about 10 and 100 times greater than those observed at sites 1, 6, and 7, at 202 and 1447 μg kg^-1^-dry, respectively.

The National Oceanic and Atmospheric Administration’s proposed guidelines on sediment quality in the national status and trends program [21] is used to assess the potential toxicity of sediments for organisms. Sediment toxicity, *T*, for biota is categorized as effects-range low (ERL) and median (ERM) when it is within the 10th and 50th percentiles, respectively, on an ordered list of concentrations in sediment found in the literature that co-occur with any biological effect. Furthermore, sediment quality is assigned to three categories according to the occurrence of acute toxicological effects: such effects occur “rarely” for *T* < ERL, “occasionally” for ERL ≤ *T* < ERM, and “frequently” for *T* ≥ ERM [21]. Evaluation of the present results according to these guidelines shows that all sampling sites provide an ERL quotient of <1.0, indicating that the PAH levels observed at all sites would rarely cause toxicological effects. It has been feared that PAH concentrations in coastal sediments would increase as a result of disturbance by the tsunami and the production of PAHs by fires when the earthquake struck. However, contrary to these expectations, no relationship was observed between PAH concentrations in coastal sediments and the degree of damage by the tsunami and fires. The apparent lack of a relationship between these factors will be discussed in later sections.

### Origins of PAHs in sediment

**Fig 2:**
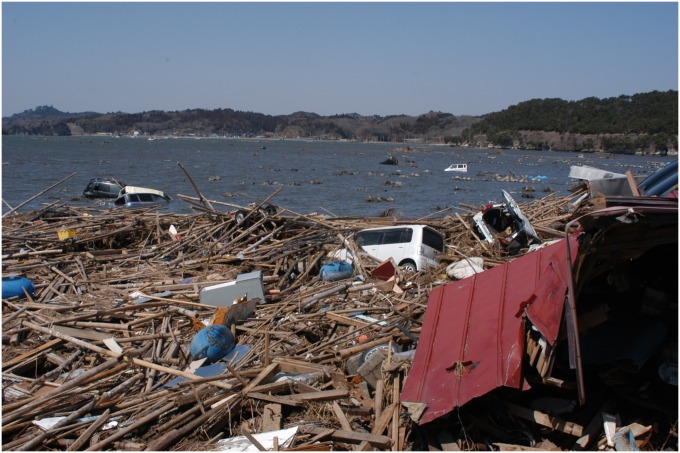
Photograph of the Tona coast at high tide on 5 April 2011. (25 days after the tsunami, showing many submerged cars).

At the time of the earthquake, dozens of vehicles were carried from the land to the shore, as at the Tona coast (site 5, Fig 2). Oil films were also frequently observed along the Tona coast following the tsunami. Therefore, it was hypothesized that oil-derived PAHs from gasoline and diesel that leaked from these vehicles would be detected in coastal sediments. Additionally, heavy oil that leaked from a damaged ship was also a candidate for the origin of the PAHs in intertidal sediments. Two ratios are commonly used to estimate PAH origin in sediments [22–24]. The [Flu]/([Flu]+[Pyr]) ratio is usually defined as the petroleum/combustion transition point, [Flu]/([Flu]+[Pyr]) < 0.5 for most petroleum samples and [Flu]/([Flu]+[Pyr]) > 0.5 for combustion samples. In addition, a ratio of [Anth]/([Anth]+[Phe]) < 0.1 mainly indicates petroleum contamination, while a ratio of [Anth]/([Anth]+[Phe]) > 0.1 is typical of a combustion source. The ratios calculated in Table 3 show that the PAHs detected at each of the sites are derived from combustion rather than oil/fuel pollution.

### PAH concentrations in bivalves

Details of the bivalves collected from the seven sites are shown in Table 2. Because it is unknown whether individual bivalves were born before or after the earthquake, we consider the bivalves used in this study to be a mixture of both. In addition, specimens of *M*. *quadrangularis* were collected in the Yoro estuary tideland, because *R*. *philippinarum* was rarely collected at this site. It is reported that these two clam species share very similar habitats and diets [25]. Therefore, in the case of the Yoro estuary tideland, *M*. *quadrangularis* was used for analysis and the PAH concentrations were compared with those of *R*. *philippinarum* collected at sites 4–6.

Table 4 shows average concentrations of individual PAHs in bivalves collected during 2013 from the Tohoku district and Tokyo Bay. Total PAH concentrations (*C*_T_) are 244, 301, and 370 μg kg^-1^-dry for clams, mussels, and oysters, respectively. The *C*_T_ values observed in bivalves from various countries (summarized in Table 5) show that the accumulation of PAHs in bivalves in the Tohoku district is comparatively low or moderate; thus, to date, pollution of the district by PAHs has not been serious.

**Table 4:** Average concentrations (μg kg^-1^-dry) of PAHs in bivalves (*n* = 3).

	Phe	Anth	Flu	Pyr	Chry	[b]flu	[a]pyr	Pery	Σ PAHs
***Clam***									
Site 4	87.6	19.4	64	53.5	30.5	26.9	33.1	26.5	342
Site 5	71.9	18.6	48.6	43.1	23.3	15.1	22.7	17.1	259
Site 6	61.2	17.4	40.6	30.4	25.2	19.3	23	24.8	241
Site 7	38.6	8.97	22.3	15.5	10.7	9.38	13.9	15.1	134
Ave.	64.8	16.1	43.9	35.6	22.4	17.7	22.9	20.9	244
***Mussel***									
Site 1	107	13.2	41.7	30.9	22	16	15.6	20.8	268
Site 2	86.5	22.3	91.7	69.1	40.2	17.8	14	9.35	351
Site 3	67.7	2.42	70.2	56.1	35.4	13.4	11	8.69	285
Ave.	87.4	19.3	67.9	52.1	32.5	15.7	13.5	13	301
***Oyster***									
Site 1	112	10.3	60.5	38.1	20.3	15.4	15.3	17.2	289
Site 2	92.9	25.8	118	83.3	53.4	29	28.8	19.2	450
Ave.	103	18	89	60.7	36.9	22.2	22.1	18.2	370

Σ PAHs is denoted by *C*_T_ in the text.

Clam: *Ruditapes philippinarum* at sites 4–6; *Mactra quadrangularis* at site 7;

Mussel: *Mytilus galloprovincialis;* Oyster: *Crassostrea gigas*

**Table 5:** Total PAH concentrations in bivalves worldwide.

Area (Country)	Species	PAH	Σ PAHs (μg kg^-1^-dry)	Ref.
***Clam***				
Jiaozhou Bay (China)	*Ruditapes philippinarum*	16	276–939	[26]
Venice lagoon (Italy)	*R*. *philippinarum*	18	34–5335	[27]
Ria Formosa Lagoon (Portugal)	*R*. *philippinarum*	16	40–1200	[28]
***Mussel***				
Tohoku district (Japan)	*Mytilus galloprovincialis*	18	129–181	[29]
Domestic (Japan)	*M*. *galloprovincialis*	18	15–526	[29]
Venice Lagoon (Italy)	*M*. *galloprovincialis*	8	67–2434	[30]
Mediterranean coast (France and Spain)	*M*. *galloprovincialis*	17	17.9–58.9	[31]
Ilha Grande Bay (Brazil)	*Perna perna*	8	3.1–17.7	[32]
Victoria Harbor (Hong Kong)	*P*. *viridis*	7	289–1640	[33]
***Oyster***				
Domestic (Japan)	*Crassostrea gigas*	18	37.5–700	[29]
Terminos Lagoon (Mexico)	*C*. *virginica*	8	154–1832	[34]
Caribbean Sea (Venezuela)	*Isognomon alatus*	6	4.16–23.8	[35]
Atlantic Ocean (USA)	*C*. *virginica*	7	2.2–135	[36]

## Discussion

### Influence of the tsunami on sediment PAH concentrations

To estimate the influence of the tsunami on *C*_T_ at the sampling sites, pre-quake *C*_T_ data are needed. As no such data are available, the concentration of [a]pyr is used as an alternative. The Ministry of the Environment collected sediments throughout Japan in 2002, and measured an average [a]pyr, *C*_P_ of 21 μg kg^-1^-dry [37]. Furthermore, Table 3 reveals a proportional relationship between total PAH concentrations (*C*_T_) and [a]pyr concentrations (R^2^ > 0.999).

A comparison of the concentrations of [a]pyr obtained in this study (Table 3), *C*_R_, with the *C*_P_ values should provide some information on the tsunami’s influence. The *C*_R_/*C*_P_ ratio calculated for sites 1–3, 6, and 7 is 0.07–0.27, and those for sites 4 and 5 were 1.1 and 8.4, respectively. It is interesting to note that the *C*_R_/*C*_P_ ratios at the former sites are < 0.3 even after the catastrophe, including at sites 2 and 3, which suffered the greatest damage. At sites 2 and 3, sediment was severely disturbed by the tsunami and there were growing concerns about the diffusion of contaminants in these areas. However, this study indicated no abnormal contamination, and found PAH levels consistent with those recorded in Japan prior to the 2011 earthquake.

On the other hand, the *C*_R_/*C*_P_ ratio of 8.4 for site 5 is very high, indicating that the total PAH concentration (*C*_T_) in 2013 was approximately 10-fold that recorded in 2002; at site 4, with a *C*_P_/*C*_R_ ratio of 1.1, the total concentration remained almost constant. Sites 4 and 5, however, were reported to have suffered little tsunami damage compared to the area along the Iwate shore. The reasons postulated for these differences in *C*_R_/*C*_P_ ratios are as follows: sites 4 and 5 are located in the innermost areas of Matsushima Bay (Fig 1), at the mouth of which lie several islands of various sizes [18]. Such geographical features should help to suppress the inundation height of the tsunami, thereby reducing the resulting tsunami damage (Table 1). It is speculated that Matsushima Bay experienced little sediment disturbance compared with sites 2 and 3, which suffered large-scale damage from the tsunami. There is another explanation why PAH concentrations at sites 2 and 3 are lower than those at sites 4 and 5. The high-energy environmental conditions at sites 2 and 3 are more likely to remove PAHs, while low energy conditions at sites 4 and 5 likely retain PAHs. Thus, the high sediment PAH concentrations at sites 4 and 5 cannot be fully explained by the degree of tsunami influence alone.

Matsushima Bay was reported to be highly polluted prior to the earthquake: [a]pyr concentrations in sediments were 440–560 μg kg^-1^-dry in 2002 [37]. Because sites 2 and 3 face the Rias coast, water depth increases considerably. Water exchange with the open ocean occurs via a narrow waterway between the islands in Matsushima Bay, and the average water depth of the bay is 5 m. This environment leads to sediment accumulation, necessitating dredging off the coast of Tona. The [a]pyr concentrations are more than 20 times higher than the national average (*C*_P_; 21 μg kg^-1^-dry), indicating that Matsushima Bay had high concentrations of sediment PAH contamination long before the earthquake. The sediment PAH concentration in 2013 was reduced to less than half that in 2002. Thus, the disruption of the sediment caused by the tsunami is likely to have removed/re-suspended the PAHs and re-deposited them across Matsushima Bay.

On the other hand, *C*_T_ concentrations at sites 1–3 are at the same low level as Tokyo Bay, but the *C*_T_ of site 1 is about half that of sites 2 and 3. The sediment PAH concentrations prior to the earthquake are unknown, and it is difficult to calculate the PAH changes caused by the tsunami with accuracy. Compared to sites 2 and 3, site 1 had a lower population density and potentially lower historical inputs from vehicle emissions, and was thus expected to have lower PAH concentrations. Given that the tsunami caused a great deal of sediment disturbance at sites 2–5, the extremely low PAH concentrations at sites 2 and 3 compared to 4 and 5 are likely due to the pre-quake PAH concentrations being historically low and high, respectively, at these sites.

From these results, there appears to be no association between the severity of the tsunami and PAH concentrations in coastal sediments. Moreover, we suggest that, for certain areas, the tsunami has had a positive influence on sediment pollution by purging PAHs from coastal sediments. However, the exact fate of these re-dispersed contaminants remains to be determined.

### Influence of seismic-induced fire on sediment PAH concentrations

As described in the introduction section, site 3 (Orikasa estuary tideland) was heavily damaged by the tsunami and by a seismic-induced fire at this locality. The fire consumed a range of fuels, including oil, domestic and automotive propane gas, and wooden structures, resulting in a burned area of approximately 17.6 ha, making it one of the largest fires caused by the 2011 earthquake [16, 17]. However, the sediment PAH concentrations at the site were almost the same as those at site 2, where no fires occurred (Table 3). Therefore, fire does not appear to have a long-term influence on PAH concentrations in sediments. This explanation is supported by the change in PAH concentrations observed in sediments of the Yoro estuary tideland after the fire. Site 6 (the Yoro estuary tideland) experienced a seismic-induced fire following the 2011 earthquake, which was started by a propane gas tank at a refinery near the estuary tideland and continued for 11 days. A month after the fire, PAH concentrations in sediment at the tidal flat were approximately 2–5 times higher than before the fire. However, these concentrations gradually declined over time, and returned to pre-fire levels after approximately 10 months [38]. Taking the scenario at site 6 into consideration, the low PAH concentrations at site 3 might be explained as follows: PAHs released from the fire spread into the sediment, resulting in temporarily elevated concentrations until a few months after the fire. The PAHs in the sediment then declined owing to various decomposition processes such as photolysis and biodegradation by benthic animals [38]. Both the speed of deposition and decomposition of PAHs were balanced by the time of our measurements in 2013, and indicate the recovery of the environment.

### Composition of PAHs in bivalves

**Fig 3:**
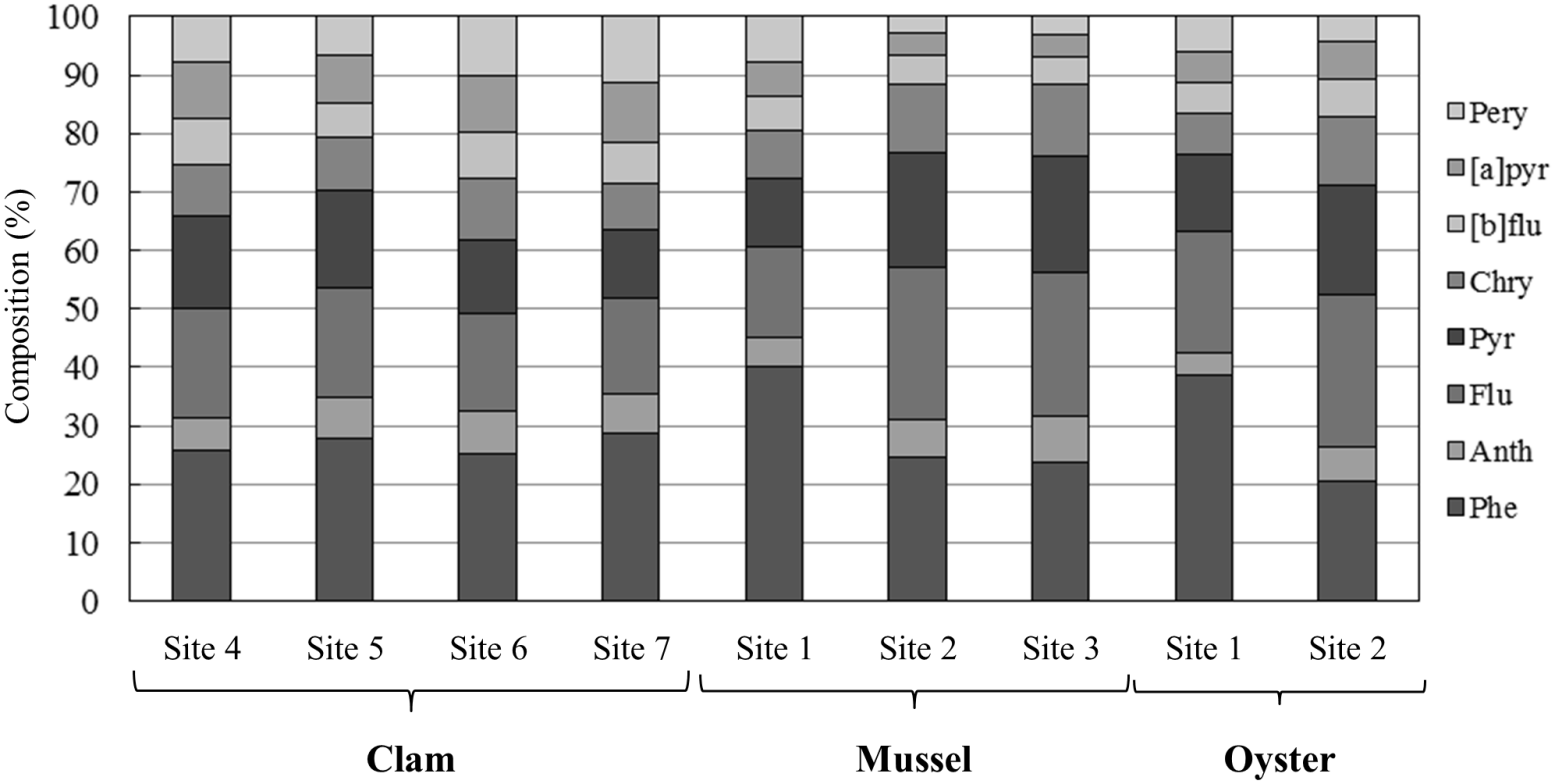
PAH compositions (%) in bivalves.

The two sampled clam species (*R*. *philippinarum* and *M*. *quadrangularis*) live in the sediment, where they feed on detritus and phytoplankton, while the surveyed mussel (*M*.*galloprovincialis*) and oyster (*C*. *gigas*) species are attached to the substrate by their byssus and left valve, respectively, and are limited to feeding on plankton. As such, the ecological niches and diets of these species differ. The PAH composition in the bivalves was dominated by Phe, Flu, and Pyr (Fig 3), which is consistent with the general tendency that bivalves are enriched in PAHs of lower molecular weight [39–42]. The results show that 2.5 years after the 2011 Japan earthquake, there was no remarkable PAH accumulation and no specific change in the PAH composition ratios was detected in the bivalves collected in 2013.

### Health risks associated with consumption of clams

Finally, we assessed the human health risks associated with dietary exposure to [a]pyr, based on the PAH concentrations in bivalves from sites 4–6. The cancer slope factor, or cancer potency, is a conservative estimate of the potential cancer risk of a contaminant, derived from dose–response data obtained from human epidemiological and animal toxicity studies. The United States Environmental Protection Agency generally uses a cancer potency factor of 7.3 (mg/kgBW–day)^-1^ for ingested [a]pyr [43], which was adopted in this study for calculating cancer risk. If a person (50 kg body weight) ingested *R*. *philippinarum* (10 g wet, which is the average per capita daily intake of clams) collected at site 4 ([a]pyr concentration 5.69 μg kg^-1^-wet), the cancer risk associated with [a]pyr is estimated using a slope factor as below:

$$5.69\;\left[\mu g\;kg^{-1}-wet\right]\times 0.01\left[kg\;day^{-1}\right]\times 7.3\left[{\left(mg/kgBW-day\right)}^{-1}\right]/50\left[kgBW\right]=8.3\times 10^{-6}$$

Based on this calculation, the carcinogenic risk from eating *R*. *philippinarum* collected at site 4 is extremely low, and is deemed safe for consumption according to the United States Environmental Protection Agency guidelines. In addition, the calculated cancer risks associated with ingesting bivalves from sites 5 and 6 are 6.3 × 10^−6^ and 6.2 × 10^−6^, respectively. These results show that there is no difference in cancer risk between the different sampling areas.

## Conclusion

The total PAH concentrations in sediments and bivalves collected in the Tohoku district of Japan in 2013 were within the ranges of 21–1447 and 134–450 μg kg^-1^-dry, respectively. Although the sampling sites were extensively damaged by the tsunami and fires in 2011, the post-disaster PAH concentrations remained low, indicating minimal influence of the tsunami on PAH pollution. Assessment of the sediments showed that contamination levels were too low to cause any effects on intertidal benthic organisms, and the concentrations present in bivalves would have negligible effects on human health through dietary intake. Interestingly, the disturbance caused by the tsunami may have purged PAHs from the sediments, forcing them below pre-quake levels in some areas.
